# Medical Gases: A Novel Strategy for Attenuating Ischemia—Reperfusion Injury in Organ Transplantation?

**DOI:** 10.1155/2012/819382

**Published:** 2012-05-07

**Authors:** Arunotai Siriussawakul, Lucinda I. Chen, John D. Lang

**Affiliations:** ^1^Department of Anesthesiology, Faculty of Medicine, Siriraj Hospital, Mahidol University, Bangkok 10700, Thailand; ^2^Department of Anesthesiology and Pain Medicine, University of Washington School of Medicine, 1959 NE Pacific Street, Seattle, WA 98195, USA

## Abstract

Ischemia reperfusion injury (IRI) is an inevitable clinical consequence in organ transplantation. It can lead to early graft nonfunction and contribute to acute and chronic graft rejection. Advanced molecular biology has revealed the highly complex nature of this phenomenon and few definitive therapies exist. This paper reviews factors involved in the pathophysiology of IRI and potential ways to attenuate it. In recent years, inhaled nitric oxide, carbon monoxide, and hydrogen sulfide have been increasingly explored as plausible novel medical gases that can attenuate IRI via multiple mechanisms, including microvascular vasorelaxation, reduced inflammation, and mitochondrial modulation. Here, we review recent advances in research utilizing inhaled nitric oxide, carbon monoxide, and hydrogen sulfide in animal and human studies of IRI and postulate on its future applications specific to solid organ transplantation.

## 1. Introduction

Organ transplantation is an established treatment that allows patients suffering from end-stage organ diseases to start living their life anew. In the United States, more than 100,000 patients are waiting for solid organ transplants, but less than 10% of patients have undergone the necessary transplantation. While the survival rate has increased substantially over the past decade, according to the U.S. Organ Procurement and Transplantation Network and the Scientific Registry of Transplant Recipients, morbidity and mortality remains substantial. With the shortage of organs, it is clear that treatments need to be developed to optimize the quality of the organs that are available and to attenuate injury to transplanted organs.

**Figure 1:**
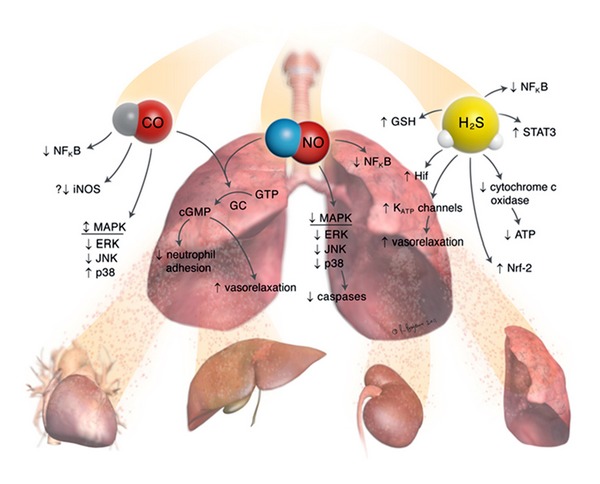
Preemptive treatment with inhaled nitric oxide, carbon monoxide and hydrogen sulfide can attenuate ischemia-reperfusion injury via modulation of a myriad of inflammatory, cellular and vascular mechanisms. GTP: guanosine triphosphate, GC: guanylate cyclase, cGMP: cyclic guanosine monophosphate, MAPK: mitogen-activated protein kinase, ERK: extracellular signal-regulated kinase, JNK: c-Jun N-terminal kinase, p38: a class of mitogen-activated complexes, iNOS: inducible nitric oxide synthase (iNOS), NFkB: nuclear factor kappa B, STAT-3: signal transducer and activator of transcription 3, ATP: adenosine triphosphate, Nrf-2: nuclear factor-like 2, Hif: hypoxia inducible factor, and GSH: glutathione.

Ischemia reperfusion injury (IRI) remains a critical clinical issue in organ transplantation. It can result in a higher incidence of acute and chronic rejection, as well as long-term morbidity and mortality [1, 2]. Ischemia is an inevitable event, starting with the cessation of arterial blood flow after organ procurement, cold ischemic time of the organ being donated, and warm ischemic time of the recipient during the organ transplantation. Reestablishment of blood flow in transplant recipients results in reperfusion injury, which is characterized by oxidative stress and inflammation (Figure 1). Much interest has been shown recently, not only in microcirculatory flow disturbances, but also in the pathophysiology of IRI in terms of the intracellular and molecular mechanisms.

A class of signaling molecules called “gasotransmitters” has been investigated as a supplementary therapeutic agent during solid organ transplantation. These medical gases, nitric oxide (NO), carbon monoxide (CO), and hydrogen sulfide (H_2_S), have traditionally been considered to be toxic and environmentally hazardous. However, numerous experimental animal and human studies of these agents have demonstrated protective effects against IRI. The aim of this review is to summarize the current understanding of medical gases on attenuation of the pathophysiology IRI in the setting of organ transplantation.

## 2. Ischemia and Reperfusion Injury in Organ Transplantation

Ischemia reperfusion injury is an occurrence where injury to an organ occurs during times of hypoxia and is amplified when blood flow (oxygen) is reestablished. Injury can be incurred during both phases of ischemia (warm and cold) [3]. Warm ischemia occurs at the time of organ procurement and reperfusion during transplantation when blood flow is restored. Cold ischemia occurs during the time of storage in preservation solution prior to the anticipated transplant [4].

Warm ischemia leads to the activation of a multitude of immunoinflammatory pathways culminating in cellular injury to a particular organ, but also systemically [5]. Warm IRI can be divided into early and late phases. The early phase occurs within the period of the first two hours of reperfusion and the late phase generally described as occurring within 6–48 hours after reperfusion. During the early phase, significant activation of immune cells occurs with resultant formation of reactive oxygen species (ROS). The late phase is characterized by neutrophil-mediated organ injury that occurs as a consequence of early phase consequences and net cumulative effect of the overlap of both phases [6].

More specifically, each organ is subjected to a particular sequence of inflammatory events leading to injury. For example in the liver, Kupffer cells, the resident macrophages appear to be instrumental in orchestrating injury during the early phase of IRI. These cells are major producers of ROS that not only leads to local injury but serves as substrate for other inflammatory reactions that inflict both local and systemic inflammatory injury. This includes cellular injury from lipid peroxidation, DNA damage, and enzyme denaturation. Generation of ROS not only leads to direct cellular injury but serves to activate other immune cell lines that hone in neutrophils. Cytokine and chemokine production occurs also during the early phase of IRI. For example, tumor necrosis factor-alpha (TNF-*α*) and interleukin-1 (IL-1) significantly increase early systemically in the serum just minutes after reperfusion. Interestingly, these cytokines along with others upregulate the production of other cytokines, chemokines, and adhesion molecules, all critical to the pattern of injury observed in the late phase of IRI [7].

Chemokines and adhesion molecules recruit neutrophils and other immune cells to the site of injury. Chemokines vital to this process include interleukin-8, and macrophage inhibitory proteins. Cells that release these molecules include resident macrophages, endothelial cells, and organ-specific parenchymal cells. Various adhesion molecules are required to the transmigration of the neutrophils journey via the bloodstream to the sites of ischemic organ injury. This is facilitated by the upregulated expression of selectins, integrins, and immunoglobulins, all which ultimately lead to the accrual of neutrophils and platelets within organ parenchyma resulting in late phase IRI [8].

Neutrophils themselves when primed by a proinflammatory stimulus are highly active and release proteases and other cytotoxic substances. Neutrophils are also responsible for the release of clinically significant concentrations of ROS through nicotinamide adenine dinucleotide phosphate hydrogen (NADPH) oxidase-dependent pathway. Activation of this enzyme system results in the predominant production of superoxide radical that in itself induces injury but also serves as substrate for a multitude of other proinflammatory reactions.

Not all of the pathways activated during the ischemia and reperfusion are injurious. Several pathways have been identified to limit injury. One of the most commonly referenced is the heme oxygenase (HO) system that is triggered due to the formation of ROS resulting in heme breakdown [9]. It is the heme breakdown products (a more neutral reduced iron), carbon monoxide (CO), and biliverdin that in their own dissimilar ways result in organ protection during IRI. The therapeutic utilization of CO will be discussed in more detail and is an obvious focus of this concise review.

Injury suffered during cold ischemia has been demonstrated to be different from warm and is directed toward injury to the endothelial cells and microcirculation as opposed to direct damage to the organ parenchymal cells. Endothelial cells seem to be more susceptible to cold storage compared to parenchymal cells [10]. Previous studies have shown that cell death is much greater in endothelial cells compared to parenchymal cells at the same time points. The injury appears to occur due to activation of apoptotic and necrotic pathways. In livers, the degree of injury has been shown to correlate with graft function following reperfusion. And overall, generalized inflammatory pathways involved in both early and late warm IRI seem to be enhanced with increasing cold storage times.

**Figure 2:**
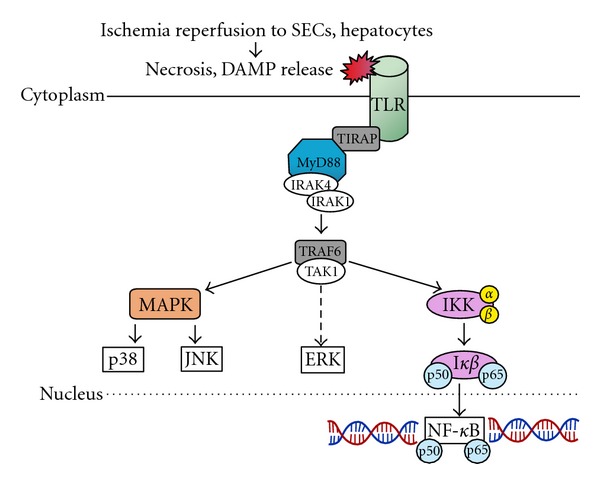
Toll-like receptor (TLR): mediated pathway during IRI. Ligation of the TLR leads to activation of upstream MyD88 and activation downstream NF-*κ*B, p38 and JNK pathways resulting in cellular injury. SEC: sinusoidal endothelial cells, DAMP: damage-associated molecular proteins [10].

The pathogenesis of IRI is also significantly influenced by toll-like receptors (TLRs) (Figure 2) [11]. These receptors are transmembrane proteins which form the major pattern recognition receptors that transduce signals in response to diverse pathogen-associated molecular patterns (PAMPs). There are a variety of TLRs (TLR 1–7,9) with each of them recognizing distinct PAMPs with their activation leading to the initiation of innate and adaptive responses through the upregulation of cytokines, chemokines, and others immune cells. Once TLRs are ligated, they undergo confirmational change and recruit cytoplasmic adapter proteins (Figure 2). The proximal adaptor proteins that mediate TLR signaling are myeloid-differentiation primary response gene 88 (MyD88). Downstream of MyD8, regulatory kinases are recruited, ultimately leading the activation of NF-*κ*B, ERK and/or activation of mitogen activation protein kinase pathways (MAPK). The net effect of activating these pathways enhanced production of inflammatory mediators thus injury.

Apoptosis or controlled program cell death is a major occurrence of IRI during organ transplantation [12]. Apoptosis pathways are activated and regulated between a multitude of signals. It has been demonstrated that organs commonly transplanted including liver, commonly incur allograft cellular apoptosis. During reperfusion both endothelial cells and parenchymal cells are susceptible to apoptosis. Many previous studies have depended on the TUNEL (terminal deoxynucleotidyl transferase-mediated dUTP nick and end labeling) assay to characterize apoptosis. This technique has been shown to stain any cell with DNA strand breaks irrespective of whether the mechanism of cell death was apoptosis or necrosis. Since the institution of more specific techniques to assist in identifying apoptotic cells, the absolute numbers of cells being affected by IRI during organ transplantation are probably not as high as previously thought. Many of the cells thought previously to have been apoptotic were probably necrotic. These necrotic cells probably lead to enhanced inflammation via activation of complement and generation of ROS. One important mediator specifically released by necrotic cells is high mobility group box 1 (HMGB1). HMGB1 is a nuclear factor bound to chromatin that during warm ischemia has been shown to bind to TLR4 and stimulate proinflammatory cytokine release. Previously, inhibition of HMGB1 attenuated cytokine release and reduced neutrophil infiltration in a model of liver IRI.

The previous paragraphs are a brief summary of the pathways involved in IRI and serve as to assist in understanding how the aforementioned inhaled medical gases may assist in attenuating IRI during organ transplantation.

## 3. Medical Gases and Organ Transplantation

The gases NO, CO, and H_2_S have been recognized as important signaling molecules. They regulate vascular tone and ameliorate inflammatory effects. Traditionally, these gases have been regarded as toxic and lethal. However, many studies have established that these gases are safe and at lower concentrations possess benefits in attenuating IRI in the setting organ transplantation. To date, there are an increasing number of reports on the role of medical gases in mitigating IRI.

## 4. NO and Organ Transplantation

Inhaled NO (iNO) was approved by the U. S. Food and Drug Administration in December 1999 for the treatment of persistent hypertension of the newborn. Over the last decade, the primary advantage of iNO has been its ability to selectively decrease pulmonary vascular resistance with minimal effects on systemic blood pressure. However, there is currently much interest in exploring its other benefits, including its antioxidant properties and cytoprotective abilities.

Nitric oxide was first identified as an endothelium-derived relaxing factor. Since then, it has been recognized for its effects as an antioxidant, an antiapoptotic agent, as well as its ability to inhibit adhesion molecules [13]. All of these properties contribute to its effectiveness in attenuating IRI. Nitrite (NO_2_^−^), which is reduced to NO during ischemia, protects mitochondria from IRI by blocking complex I of the electron transport chain, thereby limiting ROS generation and cytochrome *c* release [14]. The cytoprotective effect of nitrite in cardiac and hepatic ischemia-reperfusion in mice demonstrates the potential for nitrite, in addition to inhaled NO, to be used as treatment to modulate IRI [15]. Head-to-head prospective comparisons assessing mechanism and clinically relevant endpoints should be encouraged.

The quality and viability of an organ to be transplanted is of primary concern. With thousands of patients on organ transplantation waiting lists, it is critical to enlarge the pool of usable organs and to more predictably mitigate IRI in the donor organ. The use of nonheart-beating donors (NHBDs) would expand the donor pool, but the additional cellular damage that is incurred during warm ischemia may render these organs unusable. If however, these otherwise poorer quality organs could be salvaged, more patients could receive transplants. In a rat model of lung transplantation using NBHDs, the treatment of donors with NO during ischemia, *ex vivo* perfusion, and after transplant resulted in better oxygenation and reduced pulmonary vascular resistance [16]. A similar NO treatment could easily be translated to human transplant patients in order to see an improvement in lung function.

**Figure 3:**
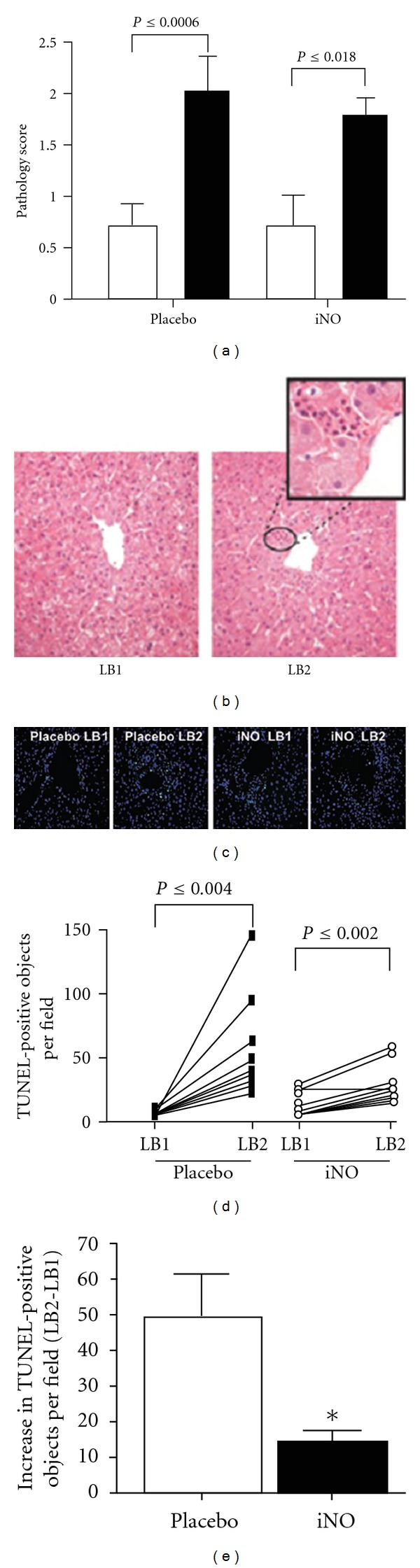
iNO decreases reperfusion-dependent hepatic cell death. (a) Histopathologic scoring of hepatic tissue samples before (white bars) and 1 hour after reperfusion (black bars). *P*-values represent significance calculated by paired *t* test. (b) Representative H&E-stained sections indicating increased injury in LB2. Original magnification ×25. The circled area is shown at a higher magnification (×100) in the inset and shows increased PMN infiltration adjacent to the hepatic vein (zone 3). (c) Representative fluorescence micrographs showing changes in TUNEL-positive cells (green); blue staining: DAPI. Original magnification, ×25. (d) Paired changes in TUNEL-positive objects in liver biopsies before (LB1) and 1 hour after reperfusion (LB2). *P* values represent significance calculated by paired *t-*test. (e) Average reperfusion-dependent increases in TUNEL-positive objects. *P ≤ 0.0005 relative to placebo [17].

Inhaled NO has been shown to be safe and efficacious in human trials. In a prospective, blinded, placebo-controlled study, 80 ppm of iNO was administered to 10 out of 20 patients undergoing orthotopic liver transplantation. Many advantages were reported in the iNO group, including reduced platelet transfusion, an improvement in the rate at which liver function was restored after transplantation, and a decrease in the length of hospital stay (Figure 3) [17]. A larger trial enrolling 80 patients is currently underway in order to confirm these results. [Add in unpublished data].

Organ rejection is a critical concern with any transplant. Inhaled NO has also been used effectively in preventing allograft dysfunction. Treatment with iNO in lung transplant patients postoperatively was shown to improve oxygenation and decrease pulmonary artery pressure. No complications were associated with the use of NO, and the mortality rate was reduced [18]. Likewise, a retrospective study of patients who received iNO before or after transplantation also presented an improvement of overall respiratory functions [19]. However, in a randomized, placebo-controlled study of 84 patients who received NO or nitrogen following lung transplantation, no differences were found between the treatment and control groups [20]. In this latter study, iNO was administered at a concentration of 20 ppm while the other two studies started at 40 ppm and gradually reduced NO concentrations. Clearly, additional prospective randomized studies are necessary to further evaluate the most effective levels of iNO usage. But it is significant to note that no adverse side effects were associated with the use of inhaled NO in any of the clinical trials [17–20].

## 5. CO and Organ Transplantation

Carbon monoxide is a lethal gas when inhaled in high concentrations for long periods of time. CO avidly binds to hemoglobin and forms carboxyhemoglobin (COHb) with an affinity more than 200 times higher than of oxygen. As a result, the blood's oxygen-carrying capacity is impaired, which eventually causes tissue hypoxia. Because of their high metabolic rate, the brain and the heart are the most sensitive to CO exposure [21].

**Figure 4:**
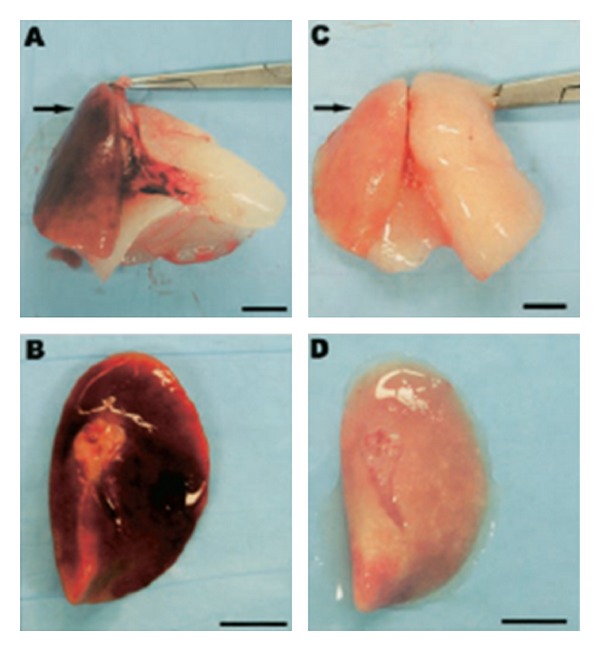
Gross anatomy of lungs from rats 6 days after transplantation (arrow in a, b) and lungs from rats 6 days after transplantation which received CO at 500 ppm. In (a) and (c), the arrow points to the left transplanted lung; the right lung is the remaining native lung. Note that in the absence of CO, transplanted lungs were noted to be markedly hyperemic (a) and (b), which was notably absent in the transplanted lung exposed to CO (c) and (d). Scale bar, 1 cm [26].

Humans and animals actually produce carbon monoxide endogenously [22]. In humans, CO arises from the action of microsomal heme oxygenase (HO) enzymes, which catalyze the conversion of heme into equimolar amounts of iron, biliverdin, and CO. HO has three isoforms: HO-1 (also known as heat shock protein 32), HO-2, and HO-3. Of the three isoforms, only HO-1 is inducible in response to a variety of cytokines and growth factors, as well as hypoxia and oxidative stress [23]. The CO that is produced by HO enzymes has multiple physiological effects, including vasodilation [24], anti-inflammatory, and antiapoptotic effects (Figure 4) [25, 26].

The known effects of carbon monoxide on the vasculature and on apoptosis make it an intriguing molecule to test in clinical models of IRI. Both inhaled CO and CO-releasing molecules (CO-RMs) have been shown to be effective in protecting rat hearts from IRI injury, resulting in less inflammation, less apoptosis, and less endothelial damage [27, 28]. Similarly, when rat livers were perfused with CO-supplemented blood or preserved in a CO-containing solution, they exhibited less hepatocyte histologic injury and a reduction in neutrophil extravasation, and they had an increased survival rate [29, 30]. There is also evidence that CO reduces delayed graft function in swine kidneys [31] and may prevent graft rejection [32].

Despite the success of CO therapy in animal studies, the utility of CO as therapy in humans is uncertain. In unpublished data, CO in Phase I trials has been shown to be well tolerated with no significant adverse effects compared to placebo [33]. Further human clinical trials should be conducted cautiously in order to determine therapeutic doses and routes of administration. In fact, a Phase II trial is in progress that investigates the ability of CO to attenuate IRI in kidney transplantation with the focus of assessing the influence of delayed graft dysfunction.

## 6. H_**2**_S and Organ Transplantation

Like NO and CO, hydrogen sulfide is primarily thought of as a toxic gas. Its reputation is well earned, for those who inhale it suffer from a range of effects, including mucosal irritation, memory loss, respiratory paralysis, and, sometimes, death. What is less known is that H_2_S naturally occurs in mammalian tissue as a byproduct of cysteine metabolism. Its production from L cysteine is catalyzed by cystathionine-*β*-synthase (CBS), cystathionine-*γ*-lyase (CSE), or 3-mercaptopyruvate sulfurtransferase (3MST). 3MST is the major H_2_S-forming enzymes in the brain while CSE is a principle H_2_S-forming enzyme in the cardiovascular system [34–36]. In other organs, such as the liver and the kidney, both enzymes contribute to H_2_S production [37].

The distinctive rotten-egg odor of H_2_S gives no indication of its therapeutic potential. Nevertheless, endogenous and exogenous H_2_S have been shown to reduce myocardial infarct size in various animal models when administered prior to reperfusion [38–42]. The cardioprotective effects are partially a result of the scavenging of hydrogen peroxide and free radicals [36, 43] and by the preservation of mitochondrial function [42]. H_2_S also attenuates IRI by dilating blood vessels through the opening of ATP-sensitive K^+^ (K_ATP_) channels in vascular smooth muscle [39, 44]. This protection against ischemic damage has been demonstrated in the liver [45], and kidney [46, 47], as well.

**Figure 5:**
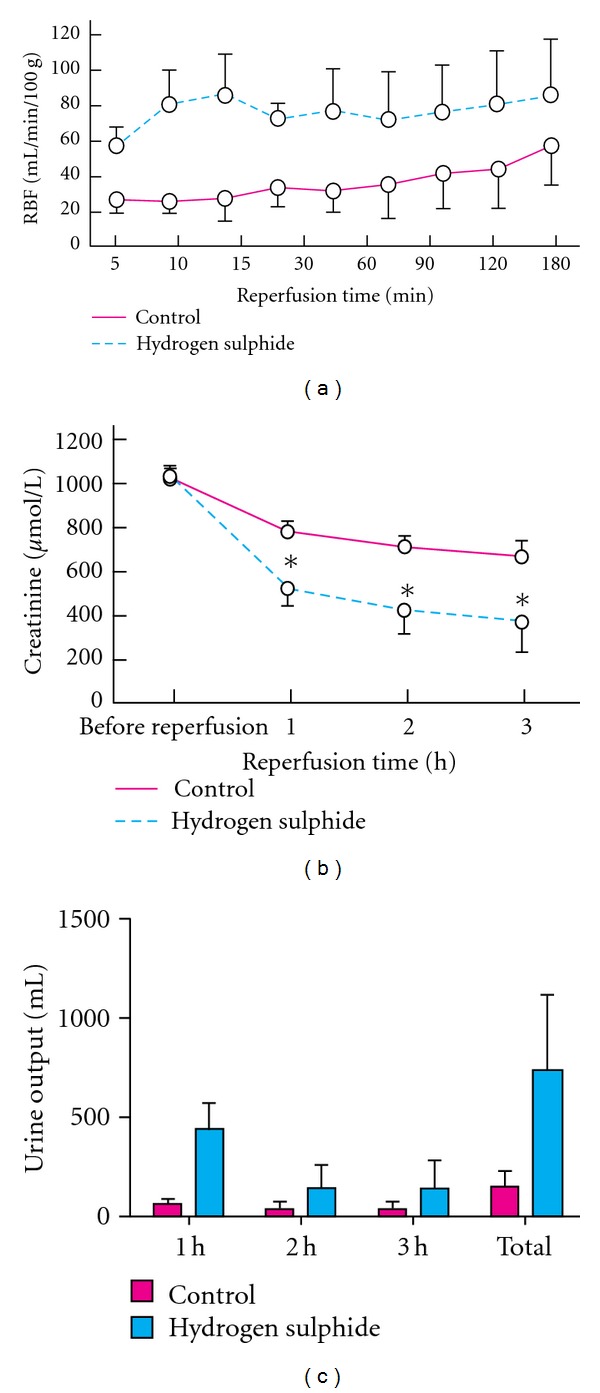
Indices of renal function were enhanced with hydrogen sulphide treatment. (a) Mean renal blood flow (RBF) over 3 hrs of reperfusion (b) Mean (s.d.) serum creatinine level over 3 hrs of reperfusion. *P < 0.050  versus control (Mann-Whitney *U* test) (c) Urine output at each hour of reperfusion [46].

With a low dose of hydrogen sulfide, mice enter a suspended-animation state with a reduced metabolic rate and lower core body temperature [48]. This artificial means of inducing a hypothermic-like state had no apparent adverse effects; thus, the potential exists to take advantage of this reduced metabolic usage to improve organ preservation. When an H_2_S donor was added to the preservation solution in an *ex vivo* rat liver model, the damaging effects of cold storage were partially reversed [49]. In a mouse model of renal transplantation, treatment with H_2_S during the hypoxic stage of IRI resulted in dramatically reduced renal damage (Figure 5) [49, 50]. The apparent mechanism for prevention of renal injury is by reversible inhibition of cytochrome *c* oxidase, the terminal enzyme of the mitochondrial electron transport chain [1, 50]. These data combined provide a compelling case for the further exploration of H_2_S usage in the attenuation and prevention of ischemia-reperfusion injury. Currently, there has not been any testing of H_2_S in human subjects, but the data from animal models is intriguing, and clinical trials are necessary to validate the efficacy of H_2_S.

## 7. Medical Gases Administered in Combination

The authors are unaware of studies performed in animals or in humans to determine the combined effects of the aforementioned gasotransmitters. It is unclear what effect combination therapy may elicit. However, both NO and CO are known to inhibit platelets by mediating a decrease in intraplatelet calcium via cGMP signaling. The effect of H_2_S on platelets has not been elucidated, but it is also believed to be a platelet inhibitor [51]. A possible complication of the use of multiple gasotransmitters during transplantation could be increased bleeding or a need for platelet transfusion during surgery. It should be noted, though, that the use of NO as a single agent decreased the frequency of platelet transfusions during liver transplantation [20], so the possibility of increased bleeding with medical gas combination treatment may be quite small.

## 8. Conclusions and Perspectives for the Future

Administration of medical gases, specifically NO, CO, and H_2_S, has been shown to mitigate IRI during organ transplantation because these gases ameliorate oxidative stress, stimulate vascular smooth muscle cell relaxation, and reduce inflammation and apoptosis. From an optimistic viewpoint, these gases would decrease acute and chronic organ rejection, thereby increasing the quality of organ transplantation. Because there are currently no other therapies being utilized that increase the quality of transplanted organs, the further testing of these gases is crucial to improving the pool of potential donor organs. However, the treatments are in their infancy, and more clinical trials are needed to determine the indications, therapeutic doses, and optimal times of administration as well as adverse effects.

## References

[1] Howard TK, Klintmalm GB, Cofer JB, Husberg BS, Goldstein RM, Gonwa TA (1990). The influence of preservation injury on rejection in the hepatic transplant recipient. *Transplantation*.

[2] Fiser SM, Tribble CG, Long SM (2002). Ischemia-reperfusion injury after lung transplantation increases risk of late bronchiolitis obliterans syndrome. *Annals of Thoracic Surgery*.

[3] Caldwell-Kenkel JC, Currin RT, Tanaka Y, Thurman RG, Lemasters JJ (1989). Reperfusion injury to endothelial cells following cold ischemic storage of rat livers. *Hepatology*.

[4] Fan C, Zwacka RM, Engelhardt JF (1999). Therapeutic approaches for ischemia/reperfusion injury in the liver. *Journal of Molecular Medicine*.

[5] Lentsch AB, Kato A, Yoshidome H, McMasters KM, Edwards MJ (2000). Inflammatory mechanisms and therapeutic strategies for warm hepatic ischemia/reperfusion injury. *Hepatology*.

[6] Yadav SS, Howell DN, Gao W, Steeber DA, Harland RC, Clavien PA (1998). L-selectin and icam-1 mediate reperfusion injury and neutrophil adhesion in the warm ischemic mouse liver. *American Journal of Physiology*.

[7] Shirasugi N, Wakabayashi G, Shimazu M (1997). Up-regulation of oxygen-derived free radicals by interleukin-1 in hepatic ischemia/reperfusion injury. *Transplantation*.

[8] Jang HR, Ko GJ, Wasowska BA, Rabb H (2009). The interaction between ischemia-reperfusion and immune responses in the kidney. *Journal of Molecular Medicine*.

[9] Öllinger R, Pratschke J (2010). Role of heme oxygenase-1 in transplantation. *Transplant International*.

[10] Teoh NC (2011). Hepatic ischemia reperfusion injury: contemporary perspectives on pathogenic mechanisms and basis for hepatoprotection-the good, bad and deadly. *Journal of Gastroenterology and Hepatology*.

[11] Malhi H, Guicciardi ME, Gores GJ (2010). Hepatocyte death: a clear and present danger. *Physiological Reviews*.

[12] Tsung A, Sahai R, Tanaka H (2005). The nuclear factor HMGB1 mediates hepatic injury after murine liver ischemia-reperfusion. *Journal of Experimental Medicine*.

[13] Phillips L, Toledo AH, Lopez-Neblina F, Anaya-Prado R, Toledo-Pereyra LH (2009). Nitric oxide mechanism of protection in ischemia and reperfusion injury. *Journal of Investigative Surgery*.

[14] Shiva S, Sack MN, Greer JJ (2007). Nitrite augments tolerance to ischemia/reperfusion injury via the modulation of mitochondrial electron transfer. *Journal of Experimental Medicine*.

[15] Duranski MR, Greer JJ, Dejam A (2005). Cytoprotective effects of nitrite during in vivo ischemia-reperfusion of the heart and liver. *Journal of Clinical Investigation*.

[16] Dong BM, Abano JB, Egan TM (2009). Nitric oxide ventilation of rat lungs from non-heart-beating donors improves posttransplant function. *American Journal of Transplantation*.

[17] Lang JD, Teng X, Chumley P (2007). Inhaled no accelerates restoration of liver function in adults following orthotopic liver transplantation. *Journal of Clinical Investigation*.

[18] Date H, Triantafillou AN, Trulock EP, Pohl MS, Cooper JD, Patterson GA (1996). Inhaled nitric oxide reduces human lung allograft dysfunction. *Journal of Thoracic and Cardiovascular Surgery*.

[19] Yerebakan C, Ugurlucan M, Bayraktar S, Bethea BT, Conte JV (2009). Effects of inhaled nitric oxide following lung transplantation. *Journal of Cardiac Surgery*.

[20] Meade MO, Granton JT, Matte-Martyn A (2003). A randomized trial of inhaled nitric oxide to prevent ischemia-reperfusion injury after lung transplantation. *American Journal of Respiratory and Critical Care Medicine*.

[21] Bauer I, Pannen BH (2009). Bench-to-bedside review: carbon monoxide—from mitochondrial poisoning to therapeutic use. *Critical Care*.

[22] Coburn RF, Blakemore WS, Forster RE (1963). Endogenous carbon monoxide production in man. *Journal of Clinical Investigation*.

[23] Wu L, Wang R (2005). Carbon monoxide: endogenous production, physiological functions, and pharmacological applications. *Pharmacological Reviews*.

[24] Lin H, McGrath JJ (1988). Vasodilating effects of carbon monoxide. *Drug and Chemical Toxicology*.

[25] Kim HS, Loughran PA, Rao J, Billiar TR, Zuckerbraun BS (2008). Carbon monoxide activates NF-kappa b via ros generation and akt pathways to protect against cell death of hepatocytes. *American Journal of Physiology*.

[26] Song R, Kubo M, Morse D (2003). Carbon monoxide induces cytoprotection in rat orthotopic lung transplantation via anti-inflammatory and anti-apoptotic effects. *The American Journal of Pathology*.

[27] Akamatsu Y, Haga M, Tyagi S (2004). Heme oxygenase-1-derived carbon monoxide protects hearts from transplant associated ischemia reperfusion injury. *The FASEB Journal*.

[28] Song H, Hoeger S, Hillebrands JL (2010). Corms protect endothelial cells during cold preservation, resulting in inhibition of intimal hyperplasia after aorta transplantation in rats. *Transplant International*.

[29] Amersi F, Shen XD, Anselmo D (2002). Ex vivo exposure to carbon monoxide prevents hepatic ischemia/reperfusion injury through p38 map kinase pathway. *Hepatology*.

[30] Ikeda A, Ueki S, Nakao A (2009). Liver graft exposure to carbon monoxide during cold storage protects sinusoidal endothelial cells and ameliorates reperfusion injury in rats. *Liver Transplantation*.

[31] Hanto DW, Maki T, Yoon MH (2010). Intraoperative administration of inhaled carbon monoxide reduces delayed graft function in kidney allografts in swine. *American Journal of Transplantation*.

[32] Martins PN, Reuzel-Selke A, Jurisch A (2005). Induction of carbon monoxide in the donor reduces graft immunogenicity and chronic graft deterioration. *Transplantation Proceedings*.

[33] Motterlini R, Otterbein LE (2010). The therapeutic potential of carbon monoxide. *Nature Reviews Drug Discovery*.

[34] Hosoki R, Matsuki N, Kimura H (1997). The possible role of hydrogen sulfide as an endogenous smooth muscle relaxant in synergy with nitric oxide. *Biochemical and Biophysical Research Communications*.

[35] Shibuya N, Tanaka M, Yoshida M (2009). 3-mercaptopyruvate sulfurtransferase produces hydrogen sulfide and bound sulfane sulfur in the brain. *Antioxidants and Redox Signaling*.

[36] Geng B, Chang L, Pan C (2004). Endogenous hydrogen sulfide regulation of myocardial injury induced by isoproterenol. *Biochemical and Biophysical Research Communications*.

[37] Stipanuk MH, Beck PW (1982). Characterization of the enzymic capacity for cysteine desulphhydration in liver and kidney of the rat. *Biochemical Journal*.

[38] Sivarajah A, McDonald MC, Thiemermann C (2006). The production of hydrogen sulfide limits myocardial ischemia and reperfusion injury and contributes to the cardioprotective effects of preconditioning with endotoxin, but not ischemia in the rat. *Shock*.

[39] Johansen D, Ytrehus K, Baxter GF (2006). Exogenous hydrogen sulfide (H_2_S) protects against regional myocardial ischemia-reperfusion injury. evidence for a role of katp channels. *Basic Research in Cardiology*.

[40] Sodha NR, Clements RT, Feng J (2008). The effects of therapeutic sulfide on myocardial apoptosis in response to ischemia-reperfusion injury. *European Journal of Cardiothoracic Surgery*.

[41] Sodha NR, Clements RT, Feng J (2009). Hydrogen sulfide therapy attenuates the inflammatory response in a porcine model of myocardial ischemia/reperfusion injury. *Journal of Thoracic and Cardiovascular Surgery*.

[42] Elrod JW, Calvert JW, Morrison J (2007). Hydrogen sulfide attenuates myocardial ischemia-reperfusion injury by preservation of mitochondrial function. *Proceedings of the National Academy of Sciences of the United States of America*.

[43] Whiteman M, Armstrong JS, Chu SH (2004). The novel neuromodulator hydrogen sulfide: an endogenous peroxynitrite ’scavenger’?. *Journal of Neurochemistry*.

[44] Zhao W, Zhang J, Lu Y, Wang R (2001). The vasorelaxant effect of H_2_S as a novel endogenous gaseous katp channel opener. *The EMBO Journal*.

[45] Kang K, Zhao M, Jiang H, Tan G, Pan S, Sun X (2009). Role of hydrogen sulfide in hepatic ischemia-reperfusion-induced injury in rats. *Liver Transplantation*.

[46] Hosgood SA, Nicholson ML (2010). Hydrogen sulphide ameliorates ischaemia-reperfusion injury in an experimental model of non-heart-beating donor kidney transplantation. *British Journal of Surgery*.

[47] Tripatara P, Patel NS, Brancaleone V (2009). Characterisation of cystathionine gamma-lyase/hydrogen sulphide pathway in ischaemia/reperfusion injury of the mouse kidney: an *in vivo* study. *European Journal of Pharmacology*.

[48] Blackstone E, Morrison M, Roth MB (2005). H_2_S induces a suspended animation-like state in mice. *Science*.

[49] Balaban CL, Rodriguez JV, Guibert EE (2011). Delivery of the bioactive gas hydrogen sulfide during cold preservation of rat liver: effects on hepatic function in an ex vivo model. *Artificial Organs*.

[50] Bos EM, Leuvenink HG, Snijder PM (2009). Hydrogen sulfide-induced hypometabolism prevents renal ischemia/reperfusion injury. *Journal of the American Society of Nephrology*.

[51] Truss NJ, Warner TD (2011). Gasotransmitters and platelets. *Pharmacology and Therapeutics*.

